# Validation of the WIMU PRO^TM^ Device for Jump Detection in Beach Volleyball: A Gender-Based Analysis during Official Competitions

**DOI:** 10.5114/jhk/196549

**Published:** 2025-05-29

**Authors:** Joaquín Martín Marzano-Felisatti, José Pino-Ortega, Antonio García-de-Alcaraz, Javier Portillo, José Francisco Guzmán-Luján, Jose Ignacio Priego-Quesada

**Affiliations:** 1 Research Group in Sports Biomechanics (GIBD), Department of Physical Education and Sports, Faculty of Physical Activity and Sport Sciences, Universitat de València, Valencia, Spain.; 2 Research Group in Sports Technique and Tactics (GITTE), Department of Physical Education and Sports, Faculty of Physical Activity and Sport Sciences, Universitat de València, Valencia, Spain.; 3 Biovetmed & Sportsci Research Group, Department of Physical Activity and Sport, Faculty of Sport Sciences, University of Murcia, San Javier, Spain.; 4 SPORT Research Group (CTS-1024), CIBIS (Centro de Investigación para el Bienestar y la Inclusión Social) Research Center, University of Almería, Almería, Spain.; 5 Faculty of Sport Sciences, Laboratory of Motor Competence and Excellence in Sport, University of Castilla-La Mancha, Toledo, Spain.

**Keywords:** sand sports, microtechnology, wearable, accelerometer, automatic detection, player load

## Abstract

Jump monitoring has become an essential procedure for training load management and injury prevention in many sports, such as beach volleyball. This study aimed to assess the validity of WIMU PRO^TM^ devices for jump detection in beach volleyball and to determine, in a preliminary way, whether gender, the player's individuality or the technical action associated with the jump could influence data accuracy. Eleven beach volleyball players (6 female and 5 male) were recorded with high-definition cameras and the WIMU PRO^TM^ device during 42 one-set official matches. The number of jumps recorded by the device was compared with the observational analysis. The instrument's sensitivity was calculated based on true positives and false positives/negatives in terms of gender, player individuality, and the type of the jump. The WIMU PRO^TM^ device presented great sensitivity (96.29%), with a lower gender difference (male = 97.20%, female = 94.56%) and higher inter-player variability in females (91.06%–98.08%) than males (95.02%–98.40%). Regarding the type of the jump, actions classified as “Others” (99.10 %) obtained the greatest sensitivity, followed by “Block” (97.25 %), “Spike” (95.75 %) and “Serve” (94.69 %). The WIMU PRO^TM^ is a valid device for automatic jump detection in beach volleyball. The variations observed in terms of gender, players' individuality, and the type of the jump highlight the importance of a context-specific individualized algorithm adjustment.

## Introduction

Beach volleyball (BV) is played on a sand surface by pair-teams (Magalhaes et al., 2011), performing power, agility, hitting and jumping actions (Batista et al., 2008; Freire et al., 2022). Amongst all game issues, the jump movement skill appears to be one of the key performance indicators from a technical-tactical point of view, as it takes part in various actions (serve, attack and block, mainly) (Freire et al., 2022), as well as from the physical perspective, where jumping quantification has great relevance in external load management (Schmidt et al., 2021). Therefore, analysis of the number of jumps performed during training and competition facilitates coaches' and trainers' decision-making in load management (Bahr and Bahr, 2014; Benson et al., 2020; Kaszuba et al., 2022; Riemann et al., 2024; Schmidt et al., 2021).

Technological developments have allowed inertial measurement units (IMUs) to automate jump counts and provide in-game real-time information (Benson et al., 2020; Villarejo-García et al., 2023). Until now, these processes have been carried out through a video observational analysis (Bahr and Bahr, 2014; Medeiros et al., 2014; Palao et al., 2015; Schmidt et al., 2021), generating valid and reliable data, but in a more time-consuming process (Schmidt et al., 2021; Skazalski et al., 2018). Therefore, IMU devices appear to be a great solution, but their reliability and validation need to be tested to confirm their accuracy (Burland et al., 2021; Gageler et al., 2015; Rantalainen et al., 2018; Skazalski et al., 2018).

Some IMU devices have been validated in different sports (Benson et al., 2020; Burland et al., 2021; Cust et al., 2021), outstanding among those being the Vert, Catapult, and WIMU PRO^TM^ devices in indoor volleyball (Villarejo-García et al., 2023). The WIMU PRO^TM^ device can use the Global Positioning System (GPS) in outdoor conditions like BV or Ultra Wave Band (UWB) sensors in indoor environments like volleyball. Although the WIMU PRO^TM^ has been validated in the laboratory (Pino-Ortega et al., 2018) and for indoor volleyball (García-de-Alcaraz et al., 2022), no reliability and validity research studies have been found for jump detection in BV competitions.

The jumping surface (rigid vs. sand) is a conditioning factor of jump movement and performance (Giatsis et al., 2004). Thus, it could affect jump detection and accuracy (Batista et al., 2008). In this sense, it is worth mentioning the relationship between the motor skill behind each jump action and the algorithm's capacity to detect it (Cust et al., 2019; García-de-Alcaraz et al., 2022; Jarning et al., 2015; Skazalski et al., 2018). Thus, factors such as gender (Bahr and Bahr, 2014; Gageler et al., 2015; Hileno et al., 2023; Palao et al., 2015), the players’ performance level (Batista et al., 2008) or role (Jarning et al., 2015; Natali et al., 2019), as well as the jump preceding the action performed in the air (serve, block or spike) may influence the accuracy of jump detection (Cust et al., 2019). For all the above reasons, this study aimed: (1) to assess the validity of WIMU PRO^TM^ devices for jump detection in BV, and (2) to determine, in a preliminary way, whether gender, the player's individuality or the move associated with the jump could influence data accuracy. It was hypothesized that (1) the WIMU PRO^TM^ device would achieve great sensitivity for jump detection in BV, and (2) the gender, the player's individuality and the technical action associated with the jump would affect device accuracy.

## Methods

### Participants

A total of 1,481 jumps were made during 42 one-set official matches, performed by 11 BV players, six women (age 27 ± 3 years, body mass 65.9 ± 3.9 kg and body height 1.73 ± 0.03 m, level Tier 4 and 3) and five men (age 28 ± 3 years, body mass 80.5 ± 4.3 kg and body height 1.84 ± 0.07 m, level Tier 4 and 3) that participated voluntarily in the study (McKay et al., 2022). All participants had no muscle-skeletal injuries at the time of testing. They signed an informed consent form giving their assent to participate. The study was conducted according to the guidelines of the Declaration of Helsinki (2013) and approved by the Ethics Committee of the University of Valencia (protocol code: 2158717; approval date: 8 September 2022).

### Technology

Players wore a tight-fit top with an interscapular compartment (vertebral T2-T4 level) where the WIMU PRO^TM^ (RealTrack Systems, Almeria, Spain) device was placed (Pino-Ortega et al., 2018). WIMU PRO^TM^ is a multi-sensor device containing four triaxial accelerometers, three triaxial gyroscopes and a triaxial manometer, as well as the GPS and the UWB (Pino-Ortega et al., 2018). These sensors generate raw data from which algorithms configured in SPRO (RealTrack Systems, Almeria, Spain) software allow automatic jump detection. In this case, the algorithm used considered the minimum take-off speed (1.4 m/s^2^), the maximum flight time (1500 ms) and the minimum landing impact force (2G) for automatic jump detection (Pino-Ortega et al., 2018).

Moreover, official matches were recorded with a GoPro Hero 4 (GoPro, Inc., San Mateo, CA, USA) high-definition camera placed at the end of the court, in the middle zone, on a 2-m high tripod for complete visibility (García-de-Alcaraz et al., 2022). High-definition videos were used for jump detection through observational analysis (Benson et al., 2020; Schleitzer et al., 2022).

### Procedures

Forty-two video recordings were uploaded and analysed separately using LINCE PLUS software through the web application (Lince Web) (Soto-Fernández et al., 2022). Observation categories were created for coding: the match number, gender (male, female), the player (assigned code) and the type of the jump (serve, spike, block, and others). Each jump was identified according to its associated move, “Serve” actions being defined as those performed to start a rally, with a jump from the back line of the court, “Spike” being a hitting jump close to the net, “Block” being a front-line defensive jump to avoid the opponent attack, and “Others” being jumps linked to other actions such as setting, dig or reception with a previous fly or movements not defined in the previous categories. Data obtained in each analysis were exported to a CSV file and unified in an Excel spreadsheet (Microsoft Corporation, Redmond, WA, USA) for further analysis.

The files generated for each WIMU PRO^TM^ device were opened in SPRO software. Data were segmented and synchronised with the corresponding match video, and the algorithm was applied for automatic jump detection, associating each jump with a video time moment. Once the data from the observational analysis and the SPRO software were obtained, a jump-by-jump comparison was made. Observational analysis was conducted by a national volleyball coach with more than ten years of coaching and video-game analysis experience. Therefore, observational analysis was considered as baseline data to examine agreement between methods as done in previous research (Benson et al., 2020; García-de-Alcaraz et al., 2022; Schleitzer et al., 2022; Skazalski et al., 2018).

For reliability in the observation, a second phase was undertaken with 15% of the sample (Tabachnick and Fidell, 1989), fifteen days after the first observation. The main observer and a second one (professor, researcher, and expert in sports performance technology with more than 30 years of experience) reviewed all the jumps to calculate the agreement between them. This analysis showed high intra-observer (*k* = 0.99) and inter-observer reliability (*k* = 0.99) using the Cohen´s Kappa coefficient (Cohen, 1960).

### Statistical Analysis

The algorithm's validity criterion was assessed by examining the instrument's sensitivity, which involved a jump-by-jump comparison of the WIMU PRO™ device with video observation as the gold standard (Gageler et al., 2015; García-de-Alcaraz et al., 2022). This comparison was based on the proportion of true positives (TP) to the sum of TP and false negatives (FN) (TP / (TP + FN)) taking into account the total number of jumps, gender, the player’s individuality, and the type of the jump (Parikh et al., 2008). The sensitivity data were complemented with the total number and the percentage of FN and false positives (FP) (Charlton et al., 2017; Schleitzer et al., 2022; Skazalski et al., 2018).

Finally, a descriptive comparative analysis between the two methods (observation vs. WIMU PRO^TM^) was carried out, considering the number of jumps made by each player over the 42 one-set matches. The number of jumps presented a non-normal distribution (Shapiro-Wilk test, *p* < 0.05). Therefore, these data were described using the median and the interquartile range. The Spearman correlation coefficient (rho) was calculated for both methods with the 95% confidence interval, and values were classified as: 1.00–0.70 (very high), 0.69–0.50 (high), 0.49–0.30 (medium), 0.29–0.10 (low) and 0.19–0.00 (no correlation). A preliminary analysis was performed using Bland-Altman plots, which were represented to show the bias (B) distribution with the upper and lower limits (± 1.96 confidence interval; U_LIM_ and L_LIM_) taking into account the total number of jumps, gender, and the type of the jump (Altman and Bland, 1983). Data analysis was performed using RStudio (version 2023.06.0, packages “irr”).

## Results

Table 1 shows the total number of jumps recorded by the observational analysis (1,481) and the WIMU PRO^TM^ device (1,426), considering gender (male = 966 vs. 939, female = 515 vs. 487) and the type of the jump (Serve = 265 vs. 251, Spike = 777 vs. 744, Block = 218 vs. 212, Others = 221 vs. 219). Sensitivity was calculated for jump detection, obtaining greater values in total jumps (96.29%), by gender (male = 97.20%, female = 94.56%), and in terms of the type of the jump (Serve = 94.69%, Spike = 95.75%, Block = 97.25%, Others = 99.10%). The total number and the percentage of FN and FP showed a gender bias towards FP in males (male = 2.5%, female = 0.6%) and FN in females (male = 2.8%, female = 5.4%). Furthermore, comparisons considering players' characteristics showed inter-player sensitivity variability ranging from 91.06% to 98.40%, as well as a personal tendency to FN (1.6%–8.9%) and FP (0.0%–5.8%).

**Table 1:** Total number of jumps in terms of gender, players, and the type of the jump.

		Observation	WIMU PRO^TM^	Height (m)	Body mass (kg)	Take-off ± SD (G)	Landing ± SD (G)	False Negative (n/%)	False Positive (n/%)	Sensitivity (%)
**Total jumps**		1481	1426	-	-	2.48 ± 1.10	5.50 ± 2.88	55 / 3.7%	27 / 1.8%	96.29
**Gender / Player**	Male	966	939	1.84	80	2.68 ± 1.24	6.19 ± 3.18	27 / 2.8%	24 / 2.5%	97.20
	Player 1	201	191	1.86	80	2.99 ± 1.21	8.68 ± 3.62	10 / 5.0%	1 / 0.5%	95.02
	Player 2	194	188	1.91	86	2.81 ± 1.52	8.18 ± 3.38	6 / 3.1%	4 / 2.1%	96.91
	Player 3	183	180	1.85	80	2.88 ± 1.18	4.74 ± 1.74	3 / 1.6 %	2 / 1.1%	98.36
	Player 4	182	179	1.71	74	2.63 ± 1.10	5.10 ± 1.82	3 / 1.6%	5 / 2.7%	98.40
	Player 5	206	201	1.85	82	2.17 ± 1.00	4.40 ± 1.87	5 / 2.4%	12 / 5.8%	97.57
	Female	515	487	1.73	66	2.06 ± 0.51	4.12 ± 1.33	28 / 5.4%	3 / 0.6 %	94.56
	Player 6	104	102	1.76	69	2.12 ± 0.56	4.48 ± 1.21	2 / 1.9%	0 / 0%	98.08
	Player 7	123	112	1.74	69	2.26 ± 0.48	3.54 ± 0.99	11 / 8.9%	0 / 0%	91.06
	Player 8	68	65	1.73	70	2.13 ± 0.39	4.66 ± 1.54	3 / 4.4%	1 / 1.5%	95.59
	Player 9	97	92	1.77	62	1.87 ± 0.65	4.59 ± 1.53	5 / 5.2%	2 / 2.1%	94.85
	Player 10	54	51	1.69	60	1.92 ± 0.28	3.99 ± 1.06	3 / 5.6 %	0 / 0%	94.44
	Player 11	69	65	1.70	65	1.96 ± 0.30	3.44 ± 1.00	4 / 5.8%	0 / 0%	94.20
**Type of the jump**										
	Serve	265	251	-	-	2.48 ± 0.82	5.99 ± 3.04	14 / 5.3%	-	94.69
	Spike	777	744	-	-	2.82 ± 1.17	5.84 ± 2.91	33 / 4.2%	-	95.75
	Block	218	212	-	-	2.18 ± 0.70	5.68 ± 2.69	6 / 2.8%	-	97.25
	Others	221	219	-	-	1.66 ± 0.88	3.57 ± 1.31	2 / 0.9%	-	99.10

Notes. False Positives and False Negatives are expressed in the total number (n) and percentages (%)

A second quantitative analysis was undertaken considering jumps performed by players in each set, and the Spearman correlation coefficient calculation allowed a between-methods agreement comparison (Table 2). The Spearman correlation values showed a very high correlation in total jumps (rho = 0.99, [0.99–1.00], *p* < 0.01), by gender (rho_MALE_ = 0.97, [0.91–0.99], *p* < 0.01; rho_FEMALE_ = 0.98, [0.96–0.99], *p* < 0.01) and regarding the type of the jump (rho_SERVE_ = 0.95, [0.86–1.00], *p* < 0.01; rho_SPIKE_ = 0.98, [0.95–0.99], *p* < 0.01: rho_BLOCK_ = 0.99, [0.96–1.0], *p* < 0.01; rho_OTHERS_ = 1.00, [0.98–1.00], *p* < 0.01). Moreover, inter-player Spearman correlation value variability was found in the players’ comparison with a slightly different tendency ranging from 0.88 to 1.00.

**Table 2:** Median jumps per set, considering gender, players, and the type of the jump.

		Observation		WIMU PRO^TM^		Spearman correlation results			
		Median	Q1–Q3	Median	Q1–Q3	rho	*p* value	CI95 lower	CI95 upper
Jumps / Set		17.00	13.00–25.00	16.00	12.00–25.00	0.99	< 0.01	0.99	1.00
Gender / Player	Male	25.00	23.00–33.50	25.00	22.50–32.50	0.97	< 0.01	0.91	0.99
	Player 1	30.00	21.50–33.00	29.00	21.50–31.00	0.88	< 0.01	0.40	1.00
	Player 2	26.00	21.50–32.00	24.00	21.50–32.50	0.96	< 0.01	0.70	1.00
	Player 3	24.50	32.00–25.75	25.00	20.75–25.75	0.96	< 0.01	0.81	1.00
	Player 4	30.50	25.75–33.75	31.00	25.75–34.75	0.94	< 0.01	0.52	1.00
	Player 5	25.00	23.50–37.00	28.00	24.50–38.00	1.00	< 0.01	1.00	1.00
	Female	14.00	10.00–15.00	13.00	9.00–15.00	0.98	< 0.01	0.96	0.99
	Player 6	14.00	8.00–15.00	13.00	7.00–15.00	0.99	< 0.01	0.93	1.00
	Player 7	13.00	11.00–17.00	12.00	9.00–16.00	1.00	< 0.01	0.93	1.00
	Player 8	12.00	8.00–14.50	12.00	8.00–13.75	1.00	< 0.01	0.76	1.00
	Player 9	14.50	12.50–20.25	14.00	12.25–19.50	1.00	< 0.01	1.00	1.00
	Player 10	11.00	9.00–15.00	10.00	8.00–15.00	0.97	< 0.01	0.55	1.00
	Player 11	14.00	14.00–14.00	13.00	12.00–14.00	0.89	0.04	0.72	1.00
Type of the jump									
	Serve	7.00	4.00–9.00	6.00	4.00–9.00	0.95	< 0.01	0.86	1.00
	Spike	10.00	7.50–13.00	9.00	7.00–13.00	0.98	< 0.01	0.95	0.99
	Block	3.00	1.00–6.00	3.00	1.00–6.00	0.99	< 0.01	0.96	1.00
	Others	3.00	2.00–6.00	3.00	2.00–6.00	1.00	< 0.01	0.98	1.00

Notes. Q1: first quartile; Q3: third quartile; rho: Spearman correlation coefficient; CI: 95% Confidence Interval (CI95)

**Figure 1:**
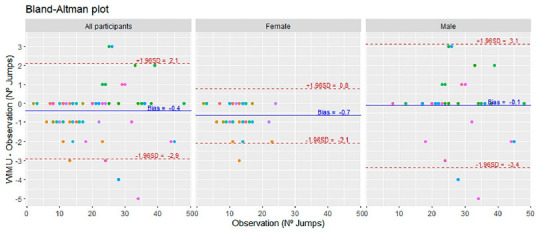
Bland-Altman plot comparing observational analysis and WIMU PRO^TM^ considering all participants and gender (female and male) with different colour distinction among players maintained throughout plots. The colour repetition represents the number of sets played by the players. The central blue line represents the absolute average difference between methods (bias), and the upper and lower discontinuous red lines represent ± 1.96 standard deviations.

**Figure 2:**
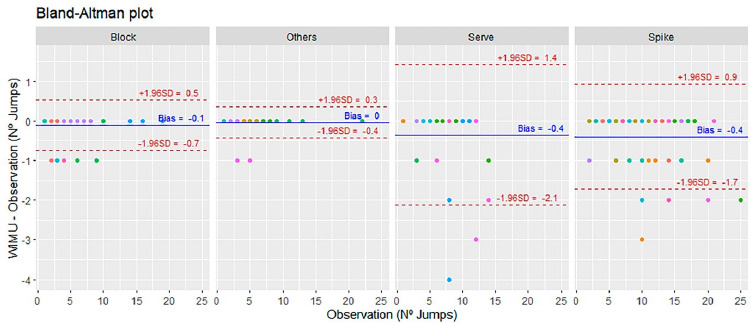
Bland-Altman plots considering the type of the jump (block, others, serve and spike) in the comparison between observational analysis and WIMU PRO^TM^ with different colour distinction among players maintained throughout plots. The colour repetition represents the number of sets played by the players where the type of the jump was made. The central blue line represents the absolute average difference between methods (bias), and the upper and lower discontinuous red lines represent ± 1.96 standard deviations.

Finally, a preliminary analysis by Blant-Altman plots showed the bias for total jumps (B_TOTAL_ = −0.4, U_LIM_ = 2.1, L_LIM_ = −2.9), gender (B_FEMALE_ = −0.7, U_LIM_ = 0.8, L_LIM_ = −2.1; B_MALE_ = −0.1, U_LIM_ = 3.1, L_LIM_ = –3.4) (Figure 1), and the type of jump comparison (B_SERVE_ = 0.36, U_LIM_ = 2.13, L_LIM_ = −1.41; B_SPIKE_ = 0.40, U_LIM_ = 1.73, L_LIM_ = −0.92; B_BLOCK_ = 0.12, U_LIM_ = 0.75, L_LIM_ = −0.52; B_OTHERS_ = 0.04, U_LIM_ = 0.42, L_LIM_ = −0.35) (Figure 2). The player’s individuality was considered by assigning a fixed colour to all plots.

## Discussion

This study aimed to assess the validity of WIMU PRO^TM^ devices for jump detection in BV, and to determine whether gender, player's individuality, or the move associated with the jump could influence data accuracy. It is important to take into account that the effect of gender was considered as a preliminary analysis to open further research, as our sample size was not sufficient to obtain definitive conclusions (e.g., six women and five men were assessed). The 96.29% total sensitivity obtained in this study is in line with findings from indoor volleyball, where accuracy and sensitivity values ranged from 96% to 99% using the VERT device (Charlton et al., 2017; MacDonald et al., 2017; Skazalski et al., 2018), 95% for GPSports (Gageler et al., 2015) and 99% for WIMU PRO^TM^ (García-de-Alcaraz et al., 2022). The WIMU PRO^TM^ superior sensitivity may be explained by the sand surface factor due to a lower capacity of players to generate ground force reactions when jumping (Giatsis et al., 2004; Schmidt et al., 2021). Moreover, the other main findings to highlight are the high Spearman correlation values obtained (rho = 0.99) and an overall average bias of −0.4 along with upper and lower standard deviation limits of 2.1 and −2.9, respectively.

As far as IMU jump validation studies for BV are concerned, only two studies have been found (Schleitzer et al., 2022; Schmidt et al., 2021), which, for the VERT system (Sports Imports, Hilliard, OH, USA), obtained accuracy of 97% (Schmidt et al., 2021), and for the MOVESENSE IMU multi-device (Suunto Oy, Finland) (chest and ankle) precision close to 96% (Schleitzer et al., 2022). It is important to mention that although the accuracy values are similar, there are some differences between devices (García-de-Alcaraz et al., 2022). The VERT system has a minimum threshold filter that removes estimated jumps below 15 cm (Skazalski et al., 2018), which may affect the detection of FP and FN. Considering the WIMU PRO^TM^ device, it was able to recognise all jumps defined as the action when a complete flight phase was produced by the athlete with both feet (Charlton et al., 2017; Skazalski et al., 2018).

The data in Table 1 examined how gender and individual player’s performance influenced the accuracy of the WIMU PRO™ device. The data showed slightly higher sensitivity for male (97.20%) compared to female players (94.56%). This small difference may be due to the different physical dynamics and jumping techniques between genders, as suggested by Gageler et al. (2015) in indoor volleyball. Additionally, the analysis of individual player data revealed variations in sensitivity, ranging from 91.06% to 98.40% among male players and 91.06% to 98.08% among female players.

Although the general female sensitivity value was lower (94.56%), the Bland-Altman plot revealed a reduced number of jumps per set, with a more homogeneous point distribution (bias near 0). No FP were detected, and a specific player's tendency (especially Players 7 and 11) to FN affected the mean bias value (−0.7), and the lower standard deviation limit (−2.1). This trend was confirmed by the FP (0.6%) and FN (5.4%), which was in line with previous research on female indoor volleyball (FP = 1%, FN = 8%) (Gageler et al., 2015). When a similar analysis was performed for the male gender, the sensitivity was slightly higher (97.20%), but the Bland-Altman plot revealed more jumps with a more heterogeneous distribution. A tendency to FP and specifically three players’ (Player 1, 2 and 3) predisposition to FN compensated the mean bias value (−0.1) and increased the upper (3.1) and lower (−3.4) standard deviation limits in comparison with females. This trend was confirmed by the FP (2.5%) and FN (2.8%) and was in line with the results of previous research carried out in men's indoor volleyball (FP = 6%, FN = 3%) (Gageler et al., 2015). All these analyses suggest that although gender effects could be discussed, individualised analyses are recommended for better automatic jump detection accuracy due to the inter-player variability (Bahr and Bahr, 2014; Jarning et al., 2015; Schleitzer et al., 2022).

Analyzing FP and FN, Table 1 data highlight significant trends related to gender and individual players. Overall, the study identified a total of 55 false negatives (3.7%) and 27 false positives (1.8%). When examining gender differences, male players exhibited fewer false negatives (2.8%) than female players (5.4%), which aligns with the higher sensitivity observed in male players. This difference might be influenced by the more pronounced and consistent jumping mechanics typically seen in male athletes. False positives, however, were slightly higher in male (2.5%) than in female players (0.6%), indicating a possible tendency to false positives in men, which could be associated with higher weight and strength in the approach steps that could result in the device recognising strong steps as jumps.

At the individual player’s level, the variability in FN and FP was notable. For example, Player 7 had the highest false negative rate (8.9%) among all participants, suggesting difficulties in the device accurately detecting her jumps, which might be attributed to her unique jump dynamics or variability in performance. In contrast, Player 4 had one of the lowest false negative rates (1.6%), indicating consistent jump detection by the WIMU PRO™ device. Regarding false positives, Player 5 showed the highest rate (5.8%), which could be due to the device mistaking other dynamic movements as jumps (strong steps and approach technique). On the other hand, several players, including Player 6, Player 7, Player 10, and Player 11, exhibited no false positives, highlighting instances where the device effectively distinguished between jumps and other movements. These findings suggest that while the WIMU PRO™ device generally performs well, individual player’s characteristics and gender-specific jumping mechanics can slightly influence the accuracy of jump detection.

Concerning movement analysis, as in previous indoor (Charlton et al., 2017; MacDonald et al., 2017; Skazalski et al., 2018) and BV studies (Schleitzer et al., 2022; Schmidt et al., 2021), sensitivity values were between 94.69% and 99.10%. Specifically, jumps defined as “Others” obtained the highest values (99.10%), followed by the “Block” (97.25%), the “Spike” (95.75%) and the “Serve” (94.69%). It can be interpreted that “Other” jumps were detected independently of players' individuality, reflecting the WIMU PRO^TM^ algorithm's capacity to detect different types of jumps, thus differing from reference studies where “Other” jumps obtained the worst precision values (41.2%) (Schmidt et al., 2021). The reason for this could be associated with the WIMU PRO^TM^ algorithm’s capacity for multi-sensor data management (Picerno et al., 2011; Spangler et al., 2018). Our sensitivity results are in line with previous research about the “Block” (TP = 97.1%, FN = 2.9%), “Spike” (TP = 96.9%, FN = 3.1%) and “Serve” (TP = 82.6%, FN = 17.4%) detection (Schleitzer et al., 2022). This tendency may be explained in terms of technical executions. Blocking is characterised by a predominantly vertical movement that facilitates automatic detection, whereas “Spike” and “Serve” make automatic detection more difficult due to individual technical adaptations (lateral adjustments and horizontal components of the movement) (García-de-Alcaraz et al., 2022; Jarning et al., 2015). Concerning “Block” jumps, these present similar values to “Other” jumps, with a mean bias = −0.1 and a 95% confidence interval with upper (0.5) and lower (−0.7) limits close to 0, thus confirming them to be one of the easiest actions to be detected (Schleitzer et al., 2022). Moreover, regarding the better capacity of “Spike” detection compared to “Serve”, this may be explained by the fact that the “Spike” action is usually maximal, and in contrast, depending on the serve execution (normally a float serve in BV) the jumps can be sub-maximal (Jarning et al., 2015; Spangler et al., 2018).

Supporting this previous information with the video-based jump-by-jump analysis, a qualitative observational section can be added to provide details related to FP and FN actions. Concerning FP jumps, they were associated with approach actions (attack, block or serve), where powerful steps were identified as jumps (horizontal components of displacement with high impacts due to impulse or changes of direction) (Jarning et al., 2015). This can explain the inter-individuality male tendency to FP in specific actions, as demonstrated in previous research (Jarning et al., 2015). Moreover, actions including falls during digs, getting out of the net, runs, abrupt direction changes or quick body projection movement while setting without sand contact loss were also associated with specific FP results (Jarning et al., 2015). Regarding FN, difficulties were found in predominantly horizontal spike actions (one-leg or lateral adjustments), blocking (lateral adjustments and getting out of the net) and serving (with quick access to the court).

Some inherent limitations are present in our work which suggests the need for further investigations. Firstly, the inability to provide the algorithm used by the company limits the study's reproducibility. Moreover, the player’s performance level could be associated with technical execution, thus, more studies in different levels of competition are needed. Our results reinforce the need for algorithm individualisation. It would be interesting to create a configurable multi-sensor algorithm with personalised calibration methods that takes into account individual biomechanical characteristics to see whether the accuracy and sensitivity of jump detection improve. Furthermore, gender comparisons should be interpreted cautiously as the sample size was small. Future research could replicate these findings with larger samples to deepen the gender comparison trends found in this study. Moreover, although this research aimed to quantify jumps in real game contexts (official competitions), future studies could consider more controlled situations, knowing a predetermined number of jumps in advance, to certify the WIMU PRO™ reliability for automated jump detection in BV.

## Conclusions

The WIMU PRO^TM^ device presents high sensitivity in detecting jump events in beach volleyball. This device enables automatic jump detection in both men's and women's categories through the algorithm provided by the manufacturer, allowing athletes, coaches, and technical staff to monitor jump load throughout the season. The player's individuality emerges as an important aspect to consider, and the “Serve” technical action is the most difficult action for automatic jump detection.
